# Synthèse, étude et validation structurale d’un triple bis-molybdate en couches, Ag_0.60_Na_0.40_Fe(MoO_4_)_2_ lié à yavapaiite

**DOI:** 10.1107/S2056989016006654

**Published:** 2016-04-26

**Authors:** Amira Souilem, Mohamed Faouzi Zid

**Affiliations:** a Laboratoire de Matériaux et Cristallochimie, Faculté des Sciences de Tunis, Université de Tunis El Manar, 2092 El Manar Tunis, Tunisia

**Keywords:** crystal structure, sodium-iron molybdate, occupational substitution

## Abstract

The title compound, Ag_0.60_Na_0.40_Fe(MoO_4_)_2_ {silver sodium iron(III) bis­[molyb­date(VI)]}, was synthesized by a solid-state reaction. The structure is built up from FeO_6_ octa­hedra and MoO_4_ tetra­hedra sharing corners to form a two-dimensional framework parallel to the (001) plane. The occupationally disordered (Ag/Na)^+^ cations are located in the inter­layer space. These [Fe(MoO_4_)_2_^−^]_*n*_ layers are similar to those in the isotypic yavapaiite-related structure of NaFe(MoO_4_)_2_. All atoms are on general positions except for Fe (site symmetry -1) and Ag/Na (site symmetry 2). The Ag/Na occupancies of the latter site were refined to 0.597 (4):0.403 (5). The title structure is compared to similar structures.

## Abstract 2

The structure of the title compound consists of a three-dimensional structure made of corner-sharing FeO_6_ octa­hedra and MoO_4_ tetra­hedra with Ag/Na cations occupying the same site in the polyhedral inter­stitial spaces.

## Contexte cristallo-chimique   

La recherche de nouveaux matériaux, pouvant être utilisés comme source d’énergie cathodique, a encouragé de nombreuses équipes de recherche de synthétiser des composés à charpente ouverte. En effet, la jonction octa­èdres–tétraèdres dans ce type de matériaux s’avère un chemin efficace pour l’élaboration de certains conducteurs ioniques: Li_3_Fe_3_(*X*O_4_)_3_ (*X* = P, As; Masquelier *et al.*, 1998 ▸), Na_7_Fe_4_(AsO_4_)_6_ (Masquelier *et al.*, 1995 ▸), LiFePO_4_ (Padhi *et al.*, 1997 ▸), KFeAs_2_O_7_ (Ouerfelli *et al.*, 2007 ▸), Ag_2_VP_2_O_8_ (Daidouh *et al.*, 1997 ▸), Na_2_CoP_2_O_7_ (Sanz *et al.*, 1999 ▸), α-Na_3_Al_2_(AsO_4_)_3_ (D’Yvoire *et al.*, 1986 ▸) et notamment la famille Nasicon Na_3_Zr_2_PSi_2_O_12_ (Goodenough *et al.*, 1976 ▸; D’Yvoire *et al.*, 1983 ▸; Boilot *et al.*, 1987 ▸).

L’élargissement des dimensions des canaux ainsi que l’occu­pation partielle des sites dans la structure devraient être deux autres facteurs favorables à la mobilité des cations monovalents. Nous avons alors choisi le molybdène (+VI) de rayon ionique supérieur à celui du silicium (+IV), du phosphore (+V) ou de l’arsenic (+V) d’après Shannon (1976 ▸) pour qu’il occupe les sites tétraédriques dans la structure. Plusieurs nouvelles phases ont été élaborées dans lesquelles les cavités octa­édriques sont occupées seulement par des ions bi- ou trivalents (Mg, Mn, Fe, Co, etc.) notamment: β-Li_0.37_Na_0.63_Fe(MoO_4_)_2_ de type wolframite (Souilem *et al.*, 2014 ▸), (Na_0.4_Li_0.6_)(Fe,Li_2_)(MoO_4_)_3_ de type lyonsite (Souilem *et al.*, 2015 ▸), KNa_5_Mn_3_(MoO_4_)_6_ (Bouzidi *et al.*, 2015 ▸) et K_0.13_Na_3.87_MgMo_3_O_12_ (Ennajeh *et al.*, 2015 ▸) de type alluaudite. Dans ce cadre un nouveau matériau de formulation Ag_0.6_Na_0.4_Fe(MoO_4_)_2_ à charpente bidimensionnelle a été élaboré par réaction à l’état solide à 1173 K. Il s’avère appartenant à la famille yavapaiite et est isostructural au composé NaFe(MoO_4_)_2_ (Klevtsova, 1975 ▸) ayant comme paramètres de maille, *a* = 9.87, *b* = 5.31, *c* = 13.57 Å, *β*=90.4° et groupe d’espace *C*2/*c*.

## Commentaire structural   

**Figure 1:**
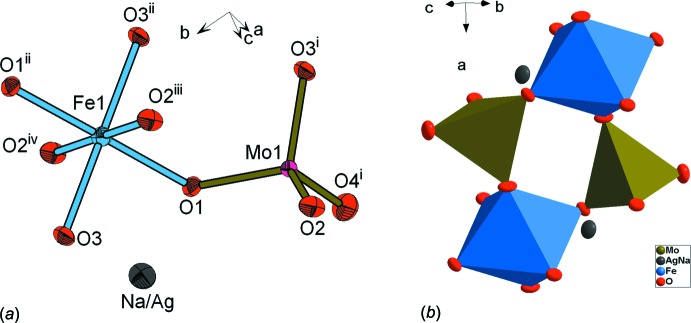
Représentation: (*a*) atomique complète des polyèdres de coordination dans l’unité asymétrique de Ag_0.60_Na_0.40_FeMo_2_O_8_; (*b*) polyédrique de la double unité Mo_2_Fe_2_O_16_. Les ellipsoides dans (*a*) ont été définis avec 50% de probabilité. [Codes de symétrie: (i) *x*, *y* − 1, *z*; (ii) −*x* + 1, −*y* + 1, −*z* + 1; (iii) −*x* + 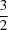, −*y* + 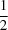, −*z* + 1; (iv) *x* − 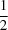, *y* + 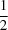, *z*.]

**Figure 2:**
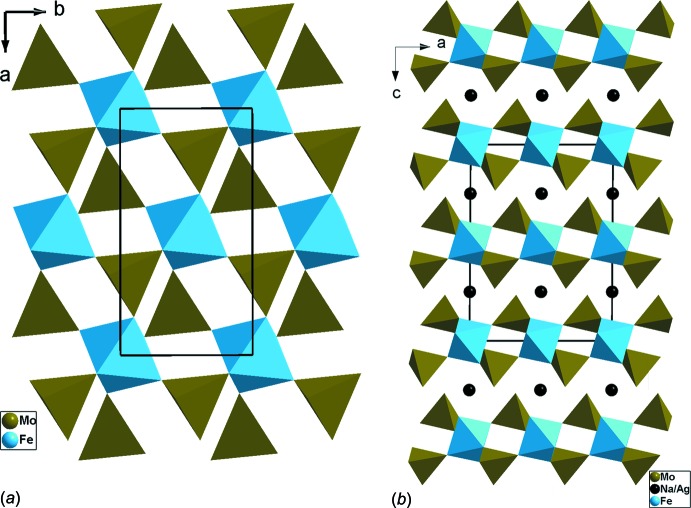
Projections: (*a*) selon l’axe *c*, d’une couche infinie de type (FeMo_2_O_8_)_*n*_ dans Ag_0.60_Na_0.40_FeMo_2_O_8_; (*b*) selon *b*, de la structure mettant en évidence l’espace inter­couches où logent les cations monovalents.

L’unité asymétrique dans Ag_0.6_Na_0.4_Fe(MoO_4_)_2_ est formée d’un tétraèdre MoO_4_ relié au moyen d’un sommet à un octa­èdre FeO_6_ et de deux cations monovalents partageant le même site cristallographique. Les polyèdres de coordination, en ajoutant les atomes d’oxygène équivalents, ainsi que les ions monovalents, sont représentés sous forme d’une unité à Fig. 1 ▸ *a*. Deux unités se regroupent par ponts mixtes de type Mo–O–Fe pour former une unité formulaire double cyclique Mo_2_Fe_2_O_16_ dans laquelle la compensation de charges est assurée par les cations Ag^+^/Na^+^ (Fig. 1 ▸ *b*). La structure peut être donc décrite moyennant des chaînes classiques de type MoFeO_8_ formées par l’association, par sommets, des unités asymétriques MoFeO_9_. Une disposition particulière, en *trans*, des tétraèdres permet la jonction des chaînes dans le plan (*ab*) et conduit à des couches (Fig. 2 ▸ *a*). Il en résulte une structure à charpente bidimens­ion­nelle où les cations Ag^+^/Na^+^ se situent dans l’espace inter­couches (Fig. 2 ▸ *b*). Les distances moyennes dans la structure étudiée pour Mo—O et Fe—O sont égales à 1.763 (3) et 1.977 93) Å, respectivement. Elles sont conformes à celles rencontrées dans les structures analogues *A*Fe(MoO_4_)_2_ (*A* = cation monovalent). Les cations Ag^+^/Na^+^ occupant les mêmes sites cristallographiques sont entourés par quatre atomes d’oxygène respectivement. La distance moyenne *A* ^+^—O (*A* ^+^ = Ag^+^/Na^+^) est égale à 2.433 (5) Å (tableau 1 ▸). Elle est de même ordre de grandeur que celles Na—O (2.426 et 2.461 Å) rencontrées respectivement dans les deux composés isotypes: NaFe(MoO_4_)_2_ (Klevtsova, 1975 ▸) et NaAl(MoO_4_)_2_ (Kolitsch *et al.*, 2003 ▸) de type yavapaiite.

Les octa­èdres FeO_6_ et les tétraèdres MoO_4_ sont relativement réguliers. Les indices de distorsion respectifs, calculés à partir des formules de Baur (1974 ▸) et Wildner (1992 ▸), sont de l’ordre de 0.6 et 1.7%. Ces grandeurs sont proches de celles calculées dans le cas de NaFe(MoO_4_)_2_, respectivement 1.6 et 4.2% pour l’octa­èdre FeO_6_ et le tétratèdre MoO_4_. La substitution partielle de l’ion Na^+^ par Ag^+^, cation polarisable, dans la structure a eu donc un léger effet sur la distorsion des polyèdres. De plus, le calcul des valences des liaisons (BVS), en utilisant la formule empirique de Brown (Brown & Altermatt, 1985 ▸) et les facteurs géométriques déduits de l’étude structurale, conduit aux valeurs des charges des ions suivants: Mo1 (5.98), Fe1 (3.12), Ag1/Na1 (1.03) ce qui confirme les degrés d’oxydation des différents ions attendus dans la structure.

La comparaison de la structure de Ag_0.6_Na_0.4_Fe(MoO_4_)_2_ avec celles rencontrées dans la littérature et construites au moyen des unités *MX*O_9_ (*M* = métal de transition et *X* = P, As ou Mo), les groupements *M*_2_ *X*_2_O_16_, les chaînes classiques *MX*O_8_, révèle une certaine filiation entre les structures de formulation analogue bien que les phases considérées présentent des symétries différentes notamment: Ba_3_Mo_2_O_2_(PO_4_)_4_, KMo(PO_4_)_2_, *A*Fe(MoO_4_)_2_ et *A*MoO_2_AsO_4_ (*A* = Li, Na ou Cs).

**Figure 3:**
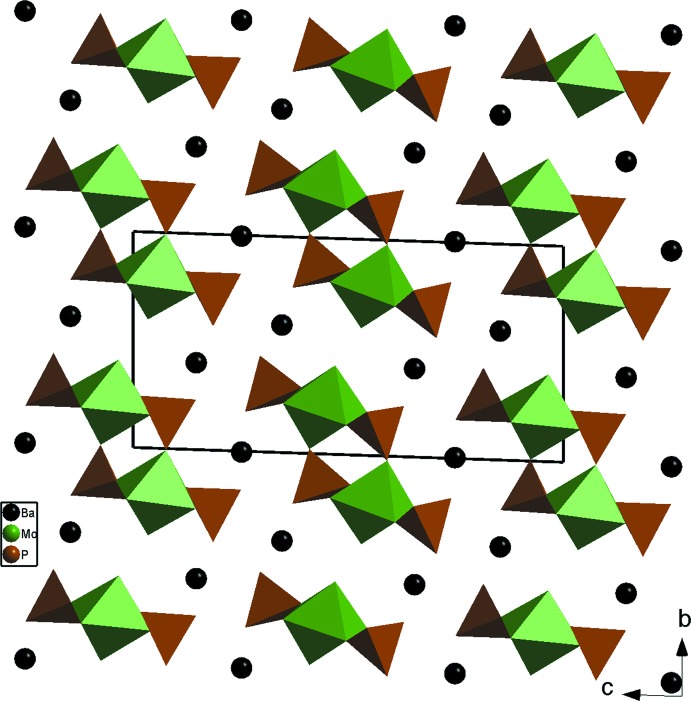
Projection de la structure de Ba_3_MoO_2_Mo_2_(PO_4_)_2_, selon *a*, mettant en évidence l’espace inter-rubans où logent les cations bivalents.

**Figure 4:**
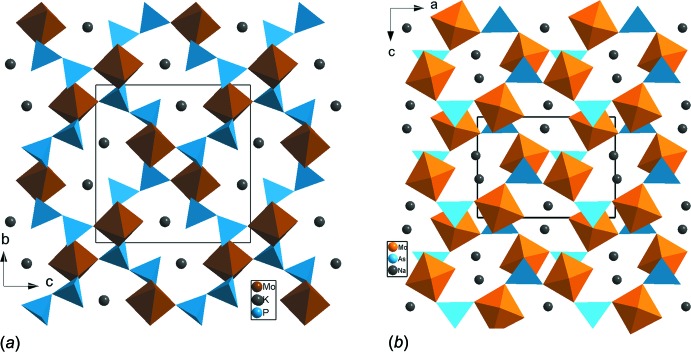
Projections de la structure: (*a*) de KMoOP_2_O_7_, selon *a*, mettant en évidence la jonction des groupements Mo_2_P_2_O_16_; (*b*) de β-NaMoO_2_AsO_4_, selon *c*, mettant en évidence la jonction des chaînes ondulées infinie MoAs_2_O_8_.

**Figure 5:**
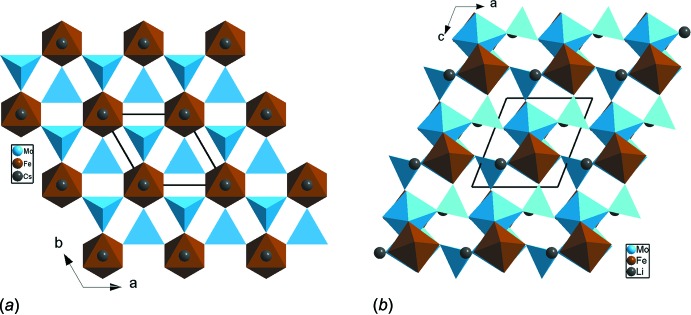
Projections de la structure de: (*a*) CsFeMo_2_O_8_, selon *c*, montrant la jonction des polyèdres par partage de sommets au sein de la couche; (*b*) LiFeMo_2_O_8_, selon *b*, montrant un autre type de couches dans lesquelles les octèdres partagent des arêtes.

En effet, dans le composé Ba_3_Mo_2_O_2_(PO_4_)_4_ (Ledain *et al.*, 1996 ▸) de symétrie triclinique (*P* 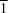) les unités MoPO_9_ se lient au moyen de ponts mixtes Mo–O–P pour former des rubans et conduisent à une charpente unidimensionnelle (Fig. 3 ▸). Dans la phase KMo(PO_4_)_2_ (Gueho *et al.*, 1992 ▸) [isotype à KNb(PO_4_)_2_; Linde *et al.*, 1980 ▸] de symétrie monoclinique, *P*2_1_/*n*) les doubles unités cycliques Mo_2_P_2_O_16_ se regroupent par partage de sommets entre tétraèdres et formation d’un molybdyldiphosphates, KMoOP_2_O_7_ (Fig. 4 ▸ *a*). Dans les deux variétés non centrosymétriques β-LiMoO_2_AsO_4_ (*P*2_1_) (Hajji *et al.*, 2004 ▸) et β-NaMoO_2_AsO_4_ (*Pca*2_1_) (Zid *et al.*, 1997 ▸), les unités MoAsO_9_ se connectent pour former des chaînes ondulées de type MoAsO_8_ qui se lient à leur tour par ponts mixtes de type Mo–O–As selon deux directions pour engendrer des charpentes tridimensionnelles possédant de larges canaux où les cations monovalents résident (Fig. 4 ▸ *b*). Le matériau étudié est un nouveau membre de la famille des bis-molybdates doubles de formulation AFe(MoO_4_)_2_ incluant LiFe(MoO_4_)_2_ (van der Lee *et al.*, 2008 ▸), NaFe(MoO_4_)_2_ (Klevtsova, 1975 ▸) et CsFe(MoO_4_)_2_ (Baza­rov *et al.*, 2010 ▸). Dans cette famille et pour les structures ayant comme groupe d’espace *P* 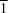 [LiFe(MoO_4_)_2_] ou *Pm* 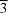 *m* [CsFe(MoO_4_)_2_], les chaînes de type FeMoO_8_ se lient d’une part par formation de ponts mixtes Fe–O–Mo (Fig. 5 ▸ *a*) et d’autre part par mise en commun d’arêtes entre les octa­èdres *M*O_6_ (*M* = Fe, Mo) et forment un autre type de couches (Fig. 5 ▸ *b*) différent de celui rencontré dans le composé étudié Ag_0.6_Na_0.4_Fe(MoO_4_)_2_.

## Synthèse et cristallisation   

Au cours de l’investigation des diagrammes *A*–Mo–Fe–O (*A* = Ag, Na) un nouveau composé de fomulation Ag_0.60_Na_0.40_Fe(MoO_4_)_2_ a été élaboré. Les cristaux ont été obtenus à partir des réactifs AgNO_3_ (Merck, 101510), Na_2_CO_3_ (Fluka, 71350), (NH_4_)_2_Mo_4_O_13_ (Fluka, 69858) et FeNO_3_·9H_2_O (Fluka 44949) pris dans les rapports molaires tels que Ag:Na:Fe:Mo restent égaux à 1:1:2:3. Le mélange a été broyé dans un mortier en agate puis placé dans un creuset en porcelaine et préchauffé à 623 K pour éliminer les produits volatils notamment: CO_2_, NO_2_, NH_3_ et H_2_O. Le résidu a été finement broyé puis remis dans le four proche de sa fusion à 1173 K. Il est maintenu à cette température pendant trois semaines pour favoriser la germination et la croissance des cristaux. Le magma final est refroidi lentement (5 K/12h) jusqu’à 1123 K puis rapidement (50 K/h) jusqu’à la température ambiante. Les cristaux de Ag_0.60_Na_0.40_Fe(MoO_4_)_2_ ont été séparés du flux à l’eau chaude. Une analyse qualitative au moyen d’un microscope électronique à balayage environnemental de type FEI Quanta 200, a confirmé la présence des éléments chimiques attendus: Ag, Mo, Fe, Na et l’oxygène.

## Résolution et affinement structural   

Un cristal sélectionné sous microscope polarisant, de bonne qualité, a servi pour la collecte des intensités (tableau 2 ▸). L’utilisation des contraintes SUMP, EADP et EXYZ autorisées par le programme *SHELX*, pour le couple d’ions Ag1/Na1, conduit à des ellipsoïdes bien définis. De plus, les densités d’électrons maximum et minimum restants dans la Fourier-différence sont acceptables et sont situées respectivement à 0.87 Å de O2 et à 0.41 Å de Fe1.

## Supplementary Material

Crystal structure: contains datablock(s) I. DOI: 10.1107/S2056989016006654/vn2110sup1.cif

Structure factors: contains datablock(s) I. DOI: 10.1107/S2056989016006654/vn2110Isup2.hkl

CCDC reference: 1423015

Additional supporting information:  crystallographic information; 3D view; checkCIF report

## Figures and Tables

**Table 1:** Longueurs de liaison sélectionnés (Å)

Mo1—O4^i^	1,711 (4)	Ag1—O4^ii^	2,499 (4)
Mo1—O3^i^	1,754 (3)	Fe1—O2^iii^	1,957 (3)
Mo1—O2	1,788 (3)	Fe1—O2^iv^	1,957 (3)
Mo1—O1	1,797 (3)	Fe1—O1	1,981 (3)
Ag1—O1	2,367 (3)	Fe1—O1^v^	1,981 (3)
Ag1—O1^ii^	2,367 (3)	Fe1—O3	1,994 (3)
Ag1—O4	2,499 (4)	Fe1—O3^v^	1,994 (3)

Codes de symétrie: (i) 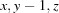; (ii) 
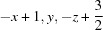
; (iii) 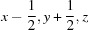; (iv) 
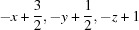
; (v) 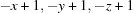.

**Table 2:** Détails expérimentaux

Données crystallines	
Formule chimique	Ag_0,60_FeMo_2_Na_0,40_O_8_
*M*_r_	449,65
Système cristallin, groupe d’espace	Monoclinique, *C*2/*c*
Température (K)	298
*a*, *b*, *c* (Å)	9,8310 (9), 5,2740 (6), 13,617 (2)
β (°)	90,334 (8)
*V* (Å^3^)	706,01 (15)
*Z*	4
Type de rayonnement	Mo *K*α
μ (mm^−1^)	7,17
Taille des cristaux (mm)	0,32 × 0,24 × 0,18

Collection de données	
Diffractomètre	Enraf–Nonius CAD-4
Correction d’absorption	ψ scan (North *et al.*, 1968 ▸)
*T*_min_, *T*_max_	0,166, 0,287
Nombre de réflexions mesurées, indépendantes et observées [*I* > 2σ(*I*)]	2875, 766, 726
*R*_int_	0,045
(sin θ/λ)_max_ (Å^−1^)	0,638

Affinement	
*R*[*F* ^2^ > 2σ(*F* ^2^)], *wR*(*F* ^2^), *S*	0,025, 0,072, 1,15
Nombre de réflexions	766
Nombre de paramètres	60
Nombre de restraints	1
Δρ_max_, Δρ_min_ (e Å^−3^)	1,36, −1,48

Programmes informatiques: *CAD-4 EXPRESS* (Duisenberg, 1992 ▸; Macíček & Yordanov, 1992 ▸), *XCAD4* (Harms & Wocadlo, 1995 ▸), *SHELXS97* et *SHELXL97* (Sheldrick, 2008 ▸), *DIAMOND* (Brandenburg & Putz, 2001 ▸), *WinGX* (Farrugia, 2012 ▸) et *publCIF* (Westrip, 2010 ▸).

## supplementary crystallographic information

### Crystal data

**Table:**

Ag_0.60_FeMo_2_Na_0.40_O_8_	*F*(000) = 826
*M**_r_* = 449.65	*D*_x_ = 4.230 Mg m^−^^3^
Monoclinic, *C*2/*c*	Mo *K*α radiation, λ = 0.71073 Å
Hall symbol: -C 2yc	Cell parameters from 25 reflections
*a* = 9.8310 (9) Å	θ = 10–15°
*b* = 5.2740 (6) Å	µ = 7.17 mm^−^^1^
*c* = 13.617 (2) Å	*T* = 298 K
β = 90.334 (8)°	Prism, green
*V* = 706.01 (15) Å^3^	0.32 × 0.24 × 0.18 mm
*Z* = 4	

### Data collection

**Table:**

Enraf–Nonius CAD-4 diffractometer	726 reflections with *I* > 2σ(*I*)
Radiation source: fine-focus sealed tube	*R*_int_ = 0.045
Graphite monochromator	θ_max_ = 27.0°, θ_min_ = 3.0°
ω/2θ scans	*h* = −12→12
Absorption correction: ψ scan (North *et al.*, 1968)	*k* = −6→6
*T*_min_ = 0.166, *T*_max_ = 0.287	*l* = −17→17
2875 measured reflections	2 standard reflections every 120 min
766 independent reflections	intensity decay: 1.2%

### Refinement

**Table:**

Refinement on *F*^2^	Primary atom site location: structure-invariant direct methods
Least-squares matrix: full	Secondary atom site location: difference Fourier map
*R*[*F*^2^ > 2σ(*F*^2^)] = 0.025	*w* = 1/[σ^2^(*F*_o_^2^) + (0.0383*P*)^2^ + 5.253*P*] where *P* = (*F*_o_^2^ + 2*F*_c_^2^)/3
*wR*(*F*^2^) = 0.072	(Δ/σ)_max_ < 0.001
*S* = 1.15	Δρ_max_ = 1.36 e Å^−^^3^
766 reflections	Δρ_min_ = −1.48 e Å^−^^3^
60 parameters	Extinction correction: *SHELXL97* (Sheldrick, 2008), Fc^*^=kFc[1+0.001xFc^2^λ^3^/sin(2θ)]^-1/4^
1 restraint	Extinction coefficient: 0.0042 (4)

### Special details

**Table:**

Geometry. All esds (except the esd in the dihedral angle between two l.s. planes) are estimated using the full covariance matrix. The cell esds are taken into account individually in the estimation of esds in distances, angles and torsion angles; correlations between esds in cell parameters are only used when they are defined by crystal symmetry. An approximate (isotropic) treatment of cell esds is used for estimating esds involving l.s. planes.
Refinement. Refinement of F^2^ against ALL reflections. The weighted R-factor wR and goodness of fit S are based on F^2^, conventional R-factors R are based on F, with F set to zero for negative F^2^. The threshold expression of F^2^ > 2sigma(F^2^) is used only for calculating R-factors(gt) etc. and is not relevant to the choice of reflections for refinement. R-factors based on F^2^ are statistically about twice as large as those based on F, and R- factors based on ALL data will be even larger.

### Fractional atomic coordinates and isotropic or equivalent isotropic displacement parameters (Å^2^)

**Table:**

	*x*	*y*	*z*	*U*_iso_*/*U*_eq_	Occ. (<1)
Mo1	0.67543 (3)	0.03643 (7)	0.61867 (3)	0.0095 (2)	
Ag1	0.5000	0.57001 (16)	0.7500	0.0214 (3)	0.597 (4)
Na1	0.5000	0.57001 (16)	0.7500	0.0214 (3)	0.403 (5)
Fe1	0.5000	0.5000	0.5000	0.0138 (3)	
O1	0.5741 (3)	0.3200 (6)	0.6162 (2)	0.0146 (6)	
O2	0.8459 (3)	0.1020 (7)	0.5812 (3)	0.0180 (7)	
O3	0.6013 (3)	0.8108 (6)	0.5404 (2)	0.0168 (7)	
O4	0.6740 (4)	0.9128 (7)	0.7348 (3)	0.0249 (8)	

### Atomic displacement parameters (Å^2^)

**Table:**

	*U*^11^	*U*^22^	*U*^33^	*U*^12^	*U*^13^	*U*^23^
Mo1	0.0100 (3)	0.0091 (3)	0.0095 (3)	0.00035 (12)	−0.00039 (14)	0.00001 (12)
Ag1	0.0298 (5)	0.0227 (5)	0.0116 (4)	0.000	0.0044 (3)	0.000
Na1	0.0298 (5)	0.0227 (5)	0.0116 (4)	0.000	0.0044 (3)	0.000
Fe1	0.0135 (5)	0.0141 (5)	0.0138 (5)	0.0005 (3)	−0.0009 (3)	−0.0013 (3)
O1	0.0173 (14)	0.0155 (15)	0.0109 (14)	0.0039 (12)	−0.0028 (11)	−0.0011 (12)
O2	0.0112 (14)	0.0208 (17)	0.0220 (16)	0.0001 (13)	0.0030 (12)	−0.0076 (14)
O3	0.0155 (14)	0.0112 (15)	0.0238 (16)	−0.0010 (13)	−0.0031 (12)	−0.0041 (13)
O4	0.0342 (19)	0.0241 (19)	0.0163 (17)	−0.0013 (16)	−0.0025 (14)	0.0070 (15)

### Geometric parameters (Å, º)

**Table:**

Mo1—O4^i^	1.711 (4)	Ag1—O4^ii^	2.499 (4)
Mo1—O3^i^	1.754 (3)	Fe1—O2^iii^	1.957 (3)
Mo1—O2	1.788 (3)	Fe1—O2^iv^	1.957 (3)
Mo1—O1	1.797 (3)	Fe1—O1	1.981 (3)
Ag1—O1	2.367 (3)	Fe1—O1^v^	1.981 (3)
Ag1—O1^ii^	2.367 (3)	Fe1—O3	1.994 (3)
Ag1—O4	2.499 (4)	Fe1—O3^v^	1.994 (3)
			
O4^i^—Mo1—O3^i^	107.33 (18)	O1—Fe1—O1^v^	180.00 (10)
O4^i^—Mo1—O2	110.48 (18)	O2^iii^—Fe1—O3	90.32 (14)
O3^i^—Mo1—O2	110.15 (15)	O2^iv^—Fe1—O3	89.68 (14)
O4^i^—Mo1—O1	109.08 (16)	O1—Fe1—O3	89.60 (13)
O3^i^—Mo1—O1	108.97 (14)	O1^v^—Fe1—O3	90.40 (13)
O2—Mo1—O1	110.75 (16)	O2^iii^—Fe1—O3^v^	89.68 (14)
O1—Mo1—Na1^vi^	116.83 (10)	O2^iv^—Fe1—O3^v^	90.32 (14)
O1—Ag1—O1^ii^	112.29 (16)	O1—Fe1—O3^v^	90.40 (13)
O1—Ag1—O4	97.21 (12)	O1^v^—Fe1—O3^v^	89.60 (13)
O1^ii^—Ag1—O4	132.89 (11)	O3—Fe1—O3^v^	180.000 (1)
O1—Ag1—O4^ii^	132.89 (11)	Mo1—O1—Fe1	127.94 (16)
O1^ii^—Ag1—O4^ii^	97.21 (12)	Mo1—O1—Ag1	128.48 (15)
O4—Ag1—O4^ii^	87.30 (18)	Fe1—O1—Ag1	103.57 (13)
O2^iii^—Fe1—O2^iv^	180.000 (1)	Mo1—O2—Fe1^vi^	147.0 (2)
O2^iii^—Fe1—O1	87.84 (14)	Mo1—O2—Na1^vi^	105.22 (15)
O2^iv^—Fe1—O1	92.16 (14)	Fe1^vi^—O2—Na1^vi^	91.76 (12)
O2^iii^—Fe1—O1^v^	92.16 (14)	Mo1^vii^—O3—Fe1	158.6 (2)
O2^iv^—Fe1—O1^v^	87.84 (14)	Mo1^vii^—O4—Ag1	111.19 (18)

Symmetry codes: (i) *x*, *y*−1, *z*; (ii) −*x*+1, *y*, −*z*+3/2; (iii) *x*−1/2, *y*+1/2, *z*; (iv) −*x*+3/2, −*y*+1/2, −*z*+1; (v) −*x*+1, −*y*+1, −*z*+1; (vi) *x*+1/2, *y*−1/2, *z*; (vii) *x*, *y*+1, *z*.
